# Engineering Strategies for Plant-Derived Extracellular Vesicles: Modification, Drug Delivery Performance, and Synergistic Effects with Gel Composite Systems

**DOI:** 10.3390/pharmaceutics18060659

**Published:** 2026-05-27

**Authors:** Xiaoxiao Qiu, Yilixiati Wusiman, Nazhakaiti Yusufujiang, Dilihuma Dilimulati, Alhar Baishan, Yipaerguli Paerhati, Alifeiye Aikebaier, Wenting Zhou

**Affiliations:** 1 Department of Pharmacology, College of Pharmacy, Xinjiang Medical University, Urumqi 830017, China; qiuxiaoxiao@stu.xjmu.edu.cn (X.Q.); yilixiati@stu.xjmu.edu.cn (Y.W.); nzkt0110@stu.xjmu.edu.cn (N.Y.); dilihuma@stu.xjmu.edu.cn (D.D.); alhar@stu.xjmu.edu.cn (A.B.); yipaerguli@stu.xjmu.edu.cn (Y.P.); alifeiye@stu.xjmu.edu.cn (A.A.); 2 Xinjiang Key Laboratory of Natural Medicines Active Components and Drug Release Technology, Urumqi 830017, China; 3 Xinjiang Key Laboratory of Biopharmaceuticals and Medical Devices, Urumqi 830017, China; 4 Engineering Research Center of Xinjiang and Central Asian Medicine Resources, Ministry of Education, Urumqi 830017, China

**Keywords:** plant-derived extracellular vesicles, drug delivery, engineered modification, gel composites, synergistic therapy, nanocarriers

## Abstract

Plant-derived extracellular vesicles (PDEVs) are a novel category of natural nanocarriers with widespread availability, low immunogenicity, high biocompatibility, and inherent pharmacological activity. These features underscore their value as dual-function systems capable of serving as both carriers and bioactive agents. Unlike previous reviews that focused primarily on disease-specific applications or on individual engineering techniques, this review established a conceptual framework integrating three interconnected dimensions: (i) engineering strategies that address the inherent limitations of PDEVs (targeting, stability, loading efficiency); (ii) the carrier-performance-synergy paradigm linking PDEV composition to therapeutic outcomes; and (iii) gel-composite design principles that transform local retention into a controllable delivery platform. This review delves into various engineering methodologies, including targeted modification, enhanced stability, and optimized drug loading, while elucidating the performance characteristics of PDEVs as drug carriers, focusing on their protective, targeting, and controlled-release properties. It notably investigates the synergistic interactions between the intrinsic bioactivity of PDEVs and the drugs they deliver. Furthermore, this review highlights advanced applications of PDEV gel composites in localized drug delivery, specifically emphasizing their clinical potential for treating dermatological conditions. Finally, it highlights the current challenges faced by PDEVs and anticipates future research directions, such as synthetic biology, multi-omics analysis, and clinical translation. This review provides a theoretical framework for the rational design and clinical translation of PDEVs. It thereby promotes their innovative development in precision nanomedicine.

## 1. Introduction

Extracellular vesicles (EVs) are naturally occurring nanomaterials characterized by a lipid bilayer encapsulation and an absence of autonomous replication capability. Their capacity to transport biologically active molecules, including lipids, proteins, and nucleic acids, and to traverse biological barriers, has garnered substantial potential in drug delivery [1,2]. PDEVs are an important type of EV. They are gaining attention as nanocarriers because their plant sources are abundant and sustainable [3,4,5]; they exhibit low immunogenicity and high biocompatibility—hereafter referred to as a favorable biocompatibility profile—along with inherent biological activity [6,7,8]. This carrier–drug synergy may further expand their applications in precision medicine [9]. However, realizing this potential requires navigating the inherent tension between PDEVs’ natural bioactivity and their suboptimal pharmacokinetic profiles for targeted delivery.

While unmodified PDEVs have these advantages, they come with several delivery-related limitations: poor targeting, a brief circulation time, and low loading efficiency for hydrophobic or anionic drugs. Engineering modification strategies have progressed. Biomaterial-based approaches have been integrated. These developments yield effective ways to address these challenges [10,11,12]. Despite significant advancements, existing reviews predominantly adopt either disease-centric taxonomies or technique-centric catalogs, leaving the engineering-design-performance causal chain insufficiently articulated. There is a notable paucity of reviews that systematically explore the rational engineering of PDEVs from a mechanistic standpoint, particularly concerning the interrelationships among vesicle composition, cellular interactions, and therapeutic efficacy. Moreover, no comprehensive review has yet established a unified design framework for PDEV–gel composite systems that correlates material properties with local delivery behavior.

This review provides a comprehensive examination of engineering strategies for PDEVs, their efficacy in drug delivery, and their synergistic interaction with gel composite materials. The analysis emphasizes the dual functionality of the carrier and its cargo, highlighting their clinical potential for localized treatment. Subsequently, this review addresses the current challenges and prospective directions for PDEVs, offering theoretical support for their rational design and clinical application, thereby fostering innovation in precision nanomedicine. By addressing this gap, we present a mechanism-driven framework that explicitly links PDEV composition to engineering rationale, delivery performance, and therapeutic synergy within integrated gel composite systems. Instead of merely cataloging applications, we propose an integrated framework that links PDEV composition, modification design, drug delivery performance, and synergistic interactions with gel systems. This work prioritizes rational design principles over descriptive enumeration, setting it apart from previous reviews by focusing on engineering logic, structure–activity relationships, and translational design principles.

## 2. Fundamental Characteristics and Separation Strategies of PDEVs

**Figure 1:**
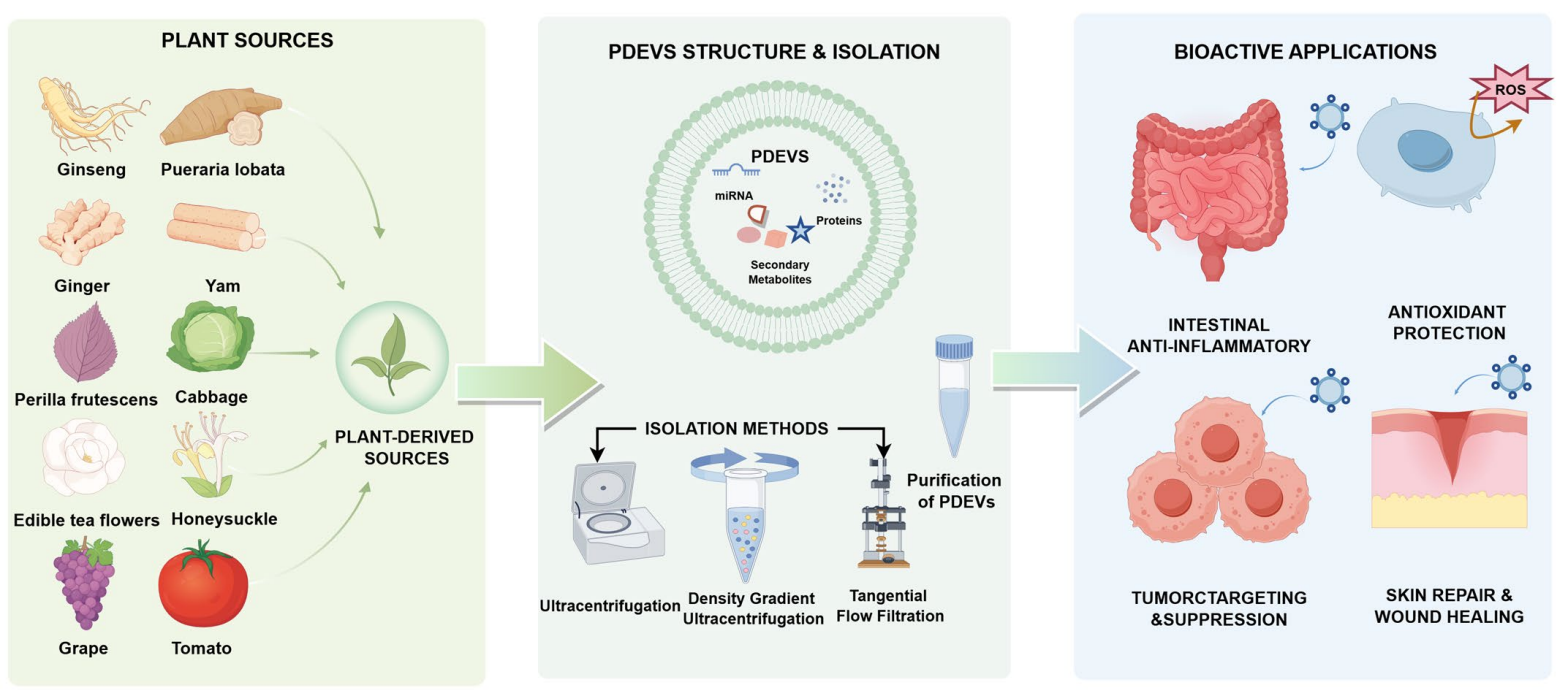
Schematic Diagram of Representative PDEVs Sources and Screening Criteria. Schematic diagram summarizing the origin, preparation, and therapeutic potential of plant-derived extracellular vesicles (PDEVs). (**Left** panel) Common edible and medicinal plants as sources of PDEVs; (**middle** panel) mainstream isolation and purification methods for PDEVs; and (**right** panel) key bioactive applications, including intestinal anti-inflammation, antioxidant protection, tumor targeting, and skin wound repair. Arrows indicate the flow of these processes and the direction of biological action.

Several intrinsic physicochemical and biological traits determine how well the PDEVs can be engineered and used for drug delivery. These features include bioactivity derived from plants, differences in composition resulting from various extraction and purification techniques, and the natural molecular structure of vesicles. These characteristics provide a solid foundation for designing engineered delivery systems. This section highlights these key features and lays the groundwork for the targeted engineering strategies discussed in the next section. Figure 1 schematically illustrates representative PDEV sources and the key screening criteria—including bioavailability, yield, biosafety, and scalability—that should guide plant selection for specific delivery applications.

### 2.1. Sources and Bioactivity

The yield, particle size, membrane composition, and bioactivity of PDEVs are highly dependent on plant source, which directly determines their suitability for drug delivery applications [13,14]. Table 1 summarizes PDEV isolation from various medicinal and edible plants, namely ginseng, ginger, *pueraria lobata,* grape, cabbage, and honeysuckle.

For targeted delivery applications, edible and medicinal plants such as ginseng, ginger, and grapes are the most promising sources. PDEVs from these species exhibit high biocompatibility, good colloidal stability, moderate particle size, and clear tissue-targeting tendencies. For example, as shown in Table 1 (entries for ginger and ginseng), although both exhibit strong anti-inflammatory activity, ginseng-derived PDEVs efficiently cross the blood–brain barrier and are suitable for neurological delivery; ginger-derived PDEVs preferentially accumulate in intestinal tissues and are ideal for oral and colonic delivery; and grape-derived PDEVs exhibit high stability and are suitable for systemic and skin delivery.

Some plant sources are less attractive for clinical translation. For instance, non-edible or non-medicinal plants may raise safety concerns for human administration. Certain herbal materials yield low amounts of vesicles, require complex purification, or produce highly heterogeneous vesicles, resulting in poor reproducibility. Plants with high lignin or cellulose content also impede efficient vesicle release and increase processing costs.

Intrinsic bioactivity serves as a key guide for source selection. Anti-inflammatory PDEVs (ginger, cabbage, honeysuckle) are preferred for inflammatory bowel disease, skin inflammation, and wound healing. Anti-tumor PDEVs (ginseng, grape, and tea flower (*Camellia sinensis*)) match cancer therapy. Osteogenic PDEVs (yam (*Dioscorea polystachya*), epimedium) are suitable for bone repair. This bioactivity–disease matching principle greatly enhances therapeutic synergy.

Yield and cost further constrain source selection. Cabbage, tomato, and grapes offer high yield and low cost, supporting large-scale production, whereas consistency in PDEV properties depends critically on cultivation region, harvest time, and processing standardization. Ginger, grapes, and cabbage are not only widely available and cost-effective but also exhibit favorable biosafety profiles, with edible-medicinal plants in particular minimizing regulatory barriers for clinical translation.

Collectively, Table 1 reveals that edible–medicinal plants with high yield, stable composition, clear bioactivity, and low cost represent the most viable sources for clinical translation. Rational source selection should balance bioactivity, physicochemical properties, yield, scalability, and biosafety to achieve optimal delivery performance.

### 2.2. Biogenesis of PDEVs

PDEVs are generated through several parallel pathways, including the multivesicular body (MVB) route, the extracellular vesicle-positive organelle (EXPO) route, and the vacuolar route [46,47,48,49]. The best understood route is the MVB pathway, which depends largely on the ESCRT complex. In this pathway, the endosomal membrane pinches inward to produce MVBs that are filled with many intraluminal vesicles. MVBs fuse with the plasma membrane and release their internal vesicles into the extracellular space. Understanding this regulation is key to developing synthetic biological strategies for PDEV production and cargo sorting [50,51,52]. The EXPO pathway uses specific organelles to directly package cytoplasmic contents into extracellular vesicles and then release them. Vesicles made this way have a membrane structure that is different from that of the MVB pathway. That affects how well they fuse with mammalian cell membranes and how stable they are as delivery vehicles [53,54]. The vacuolar pathway depends on plant-specific vacuoles to complete vesicle formation and secretion. Diverse biogenesis pathways can collectively shape membrane rigidity, surface protein composition, and luminal environment of PDEVs [55]. Elucidating these biogenic pathways helps clarify performance variations among PDEVs from different sources and batches, while laying a theoretical basis for their carrier applications.

**Figure 2:**
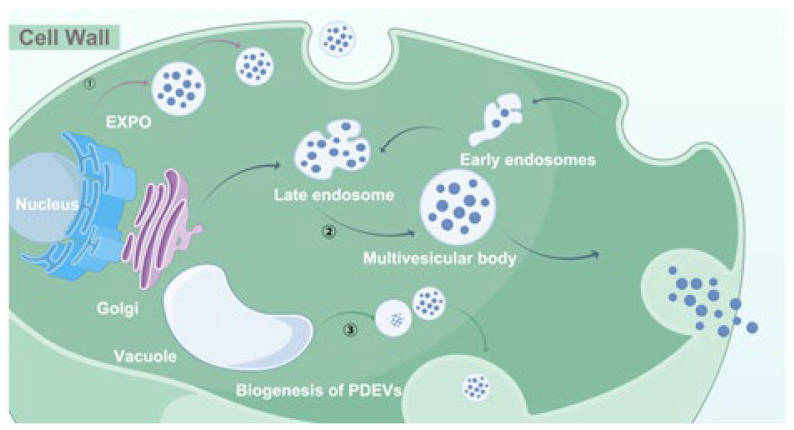
Schematic diagram of the biogenesis pathways of PDEVs. Three major routes for PDEV formation are illustrated: (①) The EXPO-mediated pathway: vesicles bud directly from the plasma membrane via the exocyst-positive organelle (EXPO) and are released into the extracellular space. (②) The endosomal sorting pathway: Early endosomes mature into late endosomes and develop into multivesicular bodies (MVBs) containing intraluminal vesicles (ILVs). MVBs subsequently fuse with the plasma membrane to secrete ILVs such as PDEVs. (③) The vacuole-derived pathway: Vesicles are generated from the vacuolar membrane and are secreted extracellularly.

The biogenesis pathways and secretion processes of PDEVs are schematically summarized in Figure 2. These three distinct biogenesis pathways give rise to PDEVs with divergent characteristics, including differences in size, membrane composition, surface protein profiles, and luminal cargo. Such intrinsic heterogeneity directly impacts the selection of appropriate separation and purification strategies. MVB-derived vesicles tend to be uniform and amenable to conventional density- or size-based purification. EXPO-derived vesicles carry unique plasma membrane-associated markers that support affinity- or charge-based purification. Vacuole-derived vesicles are often more heterogeneous and require sequential purification steps to remove plant matrix components. Understanding these biogenesis-dependent differences is therefore critical for designing rational and effective purification workflows, as discussed in Section 2.3.

### 2.3. Separation and Purification Techniques

The separation and purification of PDEVs affect the purity, activity, and efficiency of subsequent modifications. Moreover, the separation and purification of PDEVs is a mutually related and multi-step process, and its core objective is to obtain high-yield, high-purity, and fully biologically active extracellular vesicles from complex plant matrices.

Which separation method to choose usually depends on the desired yield, purity level, and specific downstream use [56]. The commonly used methods for PDEV separation include ultracentrifugation, density gradient centrifugation, microfluidic technology, immunological affinity capture, etc. Traditional methods have limitations, and new technologies are constantly being developed to strike a balance between yield, purity, and cost [57,58]. Zanotti et al. built a purification platform that combines ultrafiltration with anion exchange chromatography on a fast protein liquid chromatography system. When tested on PDEVs from Arabidopsis seedlings, this method resulted in high purity and a good yield [59].

Because each approach has its advantages and disadvantages, as compared in Table 2, UC provides high purity but low scalability, whereas TFF, combined with size exclusion chromatography (SEC) (listed in Table 2, row 6), is most suitable for clinical-grade production; a combination of strategies often overcomes these issues. In the context of fundamental mechanistic research, emphasis should be placed on purity, and it is advisable to employ combinations such as UC/DGC with SEC to ensure the reliability of the results. Conversely, for therapeutic development and clinical translation, the focus should be on scalability, cost-efficiency, and regulatory compliance. A process centered on TFF combined with SEC purification demonstrates significant potential in this regard. Although the polymer precipitation method is straightforward and rapid, the impurities it introduces limit its applicability in rigorous research settings, and it is typically utilized as a preliminary step in conjunction with other methods. Researchers should rationally select and optimize the most appropriate isolation and purification pipeline according to their research stages and specific objectives. For instance, the integration of centrifugation and UF involves initially employing differential centrifugation to eliminate impurities, thereby preventing subsequent membrane contamination and addressing challenges associated with dispersing the precipitate following direct ultra-high-speed centrifugation. Subsequently, the centrifuged product is passed through a membrane with a small aperture to concentrate the filtrate, ultimately achieving a sterilization effect. Woith et al. efficiently eliminated soluble protein contaminants by integrating density gradient centrifugation with agarose gel electrophoresis [60].

### 2.4. Characterization

Accurate characterization is essential for evaluating isolation purity, physicochemical features, and the suitability of samples for downstream functional assays. In this field, a set of standardized characterization schemes for multi-technology combinations has been formed, mainly covering three aspects: particle physical properties, morphological structure, and biochemical composition.

Systematic evaluation of the physicochemical properties of PDEVs often requires the integration of multiple complementary techniques, such as nanoparticle tracer analysis and dynamic light scattering, which are often used to measure physical properties such as particle concentration and particle size distribution. Their combined application allows mutual confirmation of size uniformity and impurity detection [72,73]. Transmission electron microscopy (TEM) allows direct observation of the morphology of typical spherical vesicles with a bilayer structure and is therefore an important tool for determining structural integrity [74,75]. The analysis of biochemical components requires the synergistic use of multiple technologies. Western blotting and enzyme-linked immunosorbent assay allow the targeted detection of specific marker proteins, such as tetraspan proteins, while heat shock protein profiling enables a comprehensive, non-targeted analysis of the proteome and lipidome. Conversely, bicinchoninic acid assay analysis and flow cytometry are ideal methods for rapid quantification of total protein mass or high-throughput characterization of surface proteins at the level of individual vesicles [76].

### 2.5. Composition

PDEVs are composed of lipids, proteins, nucleic acids, and plant-specific secondary metabolites. These components collectively underpin structural stability, cross-kingdom communication, and therapeutic efficacy. Each component class governs distinct physicochemical and biological properties—including vesicle targeting, colloidal stability, and drug loading capacity—which in turn determine cellular interactions, intracellular trafficking, and ultimate therapeutic outcomes. This composition–function framework establishes the mechanistic rationale for the rational design of PDEV-based delivery systems.

The lipid bilayer not only provides structural integrity but also governs membrane fluidity, surface charge, and hydrophobicity. These properties directly dictate nonspecific clearance, cellular internalization, and targeting efficiency. In addition, specific phospholipid species exert intrinsic anti-inflammatory activity by competing with inflammatory mediators for membrane-binding sites [77,78]; Phospholipids and sterols maintain membrane integrity and fluidity, which govern vesicle stability, cellular uptake, and endosomal escape. For example, high sterol content enhances rigidity and storage stability, while amphiphilic lipids promote interactions with target cell membranes. Studies have shown that lipid profiles differ substantially across biogenesis pathways, further influencing delivery efficiency. Moreover, lipidome remodeling under physiological stimuli enables PDEVs to function as dynamic intercellular messengers, as demonstrated by Kilasoniya et al., who showed that grapefruit-derived PDEVs enriched in unsaturated lipids significantly outperformed tomato-derived counterparts in HSP70 delivery to human cells [79].

Plant-derived microRNAs are critical mediators of cross-kingdom communication. Beyond gene regulation, microRNA profiles affect vesicle surface potential and colloidal stability, thereby influencing drug loading uniformity and intracellular retention. Upon uptake by mammalian cells, these microRNAs directly target inflammatory and oncogenic signaling pathways, amplifying therapeutic efficacy in inflammation and cancer models [80,81]. Regarding targeting, while microRNAs themselves do not mediate physical targeting, they can be exploited as therapeutic payloads that act on intracellular pathways post-delivery. For stability, microRNAs encapsulated within the aqueous lumen are protected from RNase degradation by the lipid bilayer [82]. For loading efficiency, electroporation is the most effective method for microRNA encapsulation, with optimized parameters (trehalose as a cryoprotectant, EDTA to chelate divalent cations) achieving up to 40% loading efficiency without inducing aggregation [82].

Secondary metabolites, including curcumin and baicalein, are important pharmacologically active components in PDEVs [83]. These lipophilic or amphiphilic compounds are primarily incorporated into the vesicle membrane during biogenesis or extraction via hydrophobic partitioning. Their loading efficiency depends on plant source, extraction solvent, tissue processing, and membrane permeability. Mild extraction conditions help preserve metabolite–membrane association, while harsh processing may lead to leakage. Notably, both curcumin and baicalein contribute to the anti-inflammatory and antioxidant effects of PDEVs and can be considered functional quality markers for specific plant sources.

The PDEV proteome is the most diverse, encompassing tetraspanins (CD63 homologs), heat shock proteins (HSP70 and HSP90), and plant-specific enzymes (superoxide dismutase and peroxidase) that function in vesicle biogenesis, cargo sorting, and immune modulation [81]. For targeting, surface-exposed proteins such as HSP70 and specific glycoproteins mediate recognition by C-type lectin receptors on intestinal epithelial cells and macrophages, facilitating targeted uptake without chemical modification [15]. For stability, the protein corona—the layer of proteins adsorbed onto the PDEV surface—can either stabilize vesicles by reducing aggregation or accelerate clearance by opsonization, depending on the protein composition [84]. For loading efficiency, surface proteins provide conjugation sites for ligands (e.g., RGD peptides, folate) via carbodiimide chemistry, enabling active targeting without disrupting the lipid bilayer [85,86]. A key study by Yang et al. showed that ginseng-derived PDEVs carrying specific HSP70 isoforms reprogram macrophage polarization from the M1 to M2 phenotype, reducing TNF-α production by 70% in a colitis model. Despite this progress, a unified database for PDEV marker and system proteins remains absent, hampering cross-study comparability.

**Figure 3:**
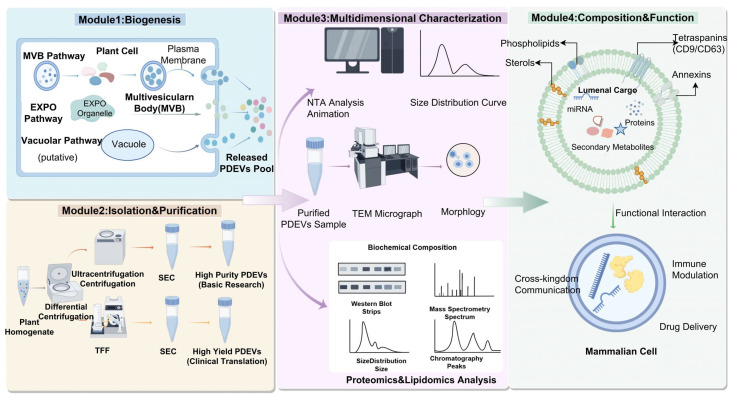
PDEVs Overview: From Occurrence to Functional Applications. This figure illustrates the complete research framework of PDEVs, encompassing four key modules: (1) Biogenesis, depicting the MVB, EXPO, and putative vacuolar pathways for PDEVs release from plant cells; (2) Isolation & Purification, which present two optimized routes for obtaining high-purity (basic research) and high-yield (clinical translation) PDEVs; (3) Multidimensional Characterization, including Nanoparticle Tracking Analysis (NTA), TEM, and omics analysis for PDEVs quality verification; and (4) Composition & Function, detailing the molecular structure of PDEVs and their core biological roles in cross-kingdom communication, immune modulation, and drug delivery.

Having detailed the sources, biogenesis pathways, isolation strategies, characterization methods, and compositional features of PDEVs, it is helpful to view these interconnected aspects as an integrated whole. The key characteristics of PDEVs discussed throughout Section 2 are visually summarized in Figure 3.

## 3. Engineering Modification Strategies for PDEVs

Although PDEVs offer many advantages as drug carriers, unmodified ones still have problems such as poor targeting, short circulation time, and low drug loading efficiency [87,88,89]. Various modification strategies have been developed to overcome these limitations. Depending on the intended goals, these approaches can be classified into three types: better targeting, greater stability, and improved drug loading. This section summarizes each strategy, including its working principles, implementation methods, and the latest research progress.

### 3.1. Targeted Modification

The goal of enhancing targeting is to guide PDEVs to reach diseased cells or tissues precisely, thereby improving their efficacy and reducing off-target toxicity. These strategies can be divided into passive and active targeting.

#### 3.1.1. Passive Targeting

Passive targeting relies on the physical and chemical properties of PDEVs and the physiological characteristics of diseased tissues to achieve targeted enrichment without additional modifications to the PDEVs. For example, research has shown that PDEVs from grapefruit juice are much better at carrying heat shock protein 70 (HSP70) and delivering it to glioma cells than PDEVs from tomato juice or other non-PDEV-mediated delivery methods. This confirms the regulatory role of PDEVs’ intrinsic membrane protein composition in passive targeting [90]. The route of administration is an indirect factor influencing passive targeting efficacy [91]. Zhuang et al. orally administered DiR-labeled ginger-derived extracellular vesicles (GDEVs). These vesicles leveraged the intestinal mucosa’s physiological properties to achieve fluorescent enrichment primarily in the liver and mesenteric lymph nodes. There was little signal enhancement in the spleen and lungs. This distribution implies that passive targeting relies on the special ill-conditioned feature of certain target tissues [92]. After intravenous injection, celery-derived EVs mainly accumulate in the liver and are ineffective at targeting lesions elsewhere due to their non-selective physical and chemical properties [93]. For solid tumors with a high degree of fibrosis and an insignificant EPR effect, most natural PDEVs have difficulty achieving effective enrichment, and their passive targeting efficiency is much lower than that of inflammatory sites, further indicating that passive targeting is highly dependent on the matching of the physiological characteristics of diseased tissues with the properties of PDEVs themselves. Although passive targeting presents benefits like operational simplicity and the preservation of the natural activity of PDEVs, it is limited by challenges such as inadequate specificity and targeting efficiency, which are contingent upon the tumor microenvironment. Consequently, it falls short of fulfilling the clinical requirements for precise delivery. Therefore, further optimization through active targeting modifications is necessary [94,95,96].

#### 3.1.2. Active Targeting

Active targeting achieves precise binding by displaying ligands on the PDEV surface that specifically recognize target cell receptors. Primary implementation strategies include chemical coupling or membrane-anchoring techniques to integrate targeting molecules like folate, arginine-glycine-aspartic acid (RGD) peptides, antibodies, and aptamers onto the PDEV membrane surface [86]. For example, modifying GDEVs with folate significantly enhances their targeting and killing ability against colon cancer cells expressing folate receptors [97]. Similarly, by conjugating heparin-binding RGD cyclic peptides targeting integrins to the surface of lemon-derived extracellular vesicles, the constructed composite carrier demonstrated significantly enhanced uptake efficiency and antitumor efficacy against ovarian cancer cells after loading with doxorubicin (DOX), while exhibiting favorable biosafety [88]. Another study incorporated PD-L1 antibodies onto the membrane surface of nanovesicles derived from *Glycyrrhiza uralensis Fisch* root-derived nanovesicles. Through the specific binding of PD-L1 antibodies to PD-L1 on tumor cell surfaces, this approach enables selective targeting of tumor cells [98]. Modification of the AS1411 aptamer, which selectively targets nucleolin that is abundantly expressed on tumor cell surfaces, onto the membrane of aloe-derived extracellular vesicles, followed by indocyanine green (ICG) loading, enables tumor-specific targeting and photothermal therapy [99]. These studies collectively demonstrate the universality of ligand engineering in precision delivery. The membrane-anchoring platform developed by Antes et al. provides a universal strategy for the standardized functional modification of PDEVs [100]. Additionally, responsive design serves as a key strategy for PDEVs to actively target modifications, primarily categorized into endogenous and exogenous types. Endogenous responsive design encompasses pH, reactive oxygen species (ROS), glutathione (GSH), and enzyme responses to trigger drug release by using the characteristics of the lesion microenvironment [86,101,102]; Exogenous responsive design achieves targeted drug delivery by integrating stimulus-responsive materials, such as photosensitizers, thermosensitizers, and sonosensitizers, into PDEVs [103,104,105,106]. These strategies have been extensively validated and applied in nanodelivery systems, providing important insights for the engineering design of PDEVs. In the field of PDEVs, such endogenous response strategies have gradually been explored. Lu et al. constructed an innovative ROS-responsive system by conjugating DBCO-NHS to the surface of Exocarpium Citri grandis-derived extracellular vesicles. In the ROS-enriched microenvironment, ROS-N_3_ undergoes hydrolysis, and the subsequent N_3_-DBCO click chemistry reaction enables targeted accumulation of Exocarpium Citri grandis-derived EVs at the lesion site [107].

Passive targeting relies on the pathophysiological features of lesions and intrinsic physicochemical properties of PDEVs without extra modification. It features simple operation and intact bioactivity but suffers from low specificity and weak enrichment in tumors with poor EPR effects. In contrast, active targeting achieves precise delivery by decorating specific ligands on the PDEV surface. Although active targeting improves specificity and cellular uptake, it may increase preparation complexity and potentially affect vesicle stability. Responsive strategies further spatiotemporally control targeting and release but face challenges regarding sensitivity, uniformity, and in vivo reproducibility.

### 3.2. Enhanced Stability

Unmodified PDEVs rapidly degrade in vivo because of their recognition and clearance by the mononuclear phagocyte system (MPS). The key to enhancing their stability is reducing non-specific interactions with the biological environment.

#### 3.2.1. Surface Engineering Methods

Surface engineering modulates PDEV membrane properties via physical or chemical modification, improving vesicle stability and mitigating non-specific interactions and rapid clearance in complex biological environments. For example, covalent conjugation or membrane insertion of PEG chains into PDEVs markedly reduces protein adsorption and recognition by immune cells. PEGylated *Asparagus cochinchinensis*-derived extracellular vesicles (ACNVs) exhibited a more than twofold enhancement in both fluorescence intensity and blood retention time compared to unmodified counterparts. Compared to linear PEG of equivalent molecular weight, branched PEG offers denser steric shielding, thereby providing superior stealth properties in vivo. However, repeated administration may induce the production of anti-PEG antibodies, which can expedite the clearance of PEG-modified vesicles from the bloodstream [108,109]. Another study showed that surface modification improved vesicle stability and therapeutic efficacy. Guan et al. conjugated cyclic RGD (cRGD) to mulberry leaf–derived extracellular vesicles (MLEs) and loaded the engineered vesicles with a urokinase-type plasminogen activator (uPA) for targeted thrombolytic therapy. These modified MLEs showed strong thrombus-homing ability and reduced non-specific clearance in vivo, allowing the encapsulated uPA to circulate longer. These vesicles ultimately achieved nearly complete thrombolysis in a femoral vein thrombosis model [110].

#### 3.2.2. Lipid Bilayer Engineering

This strategy transforms the vesicle membrane itself by means of membrane surface modification, biomimetic design-mediated membrane fusion, or mixed membrane construction, which not only improves stability but also endows vesicles with low immunogenicity, prolonged circulation time, and enhanced targeting ability [107,111,112]. For instance, fusing bacterial membrane vesicles with spinach thylakoid nanovesicles generates bacterial-plant hybrid membrane vesicles (BPNs) that carry the biological features of both parent vesicle types. These hybrid vesicles exhibit strong tumor tropism and sustained targeted accumulation. Their optimized membrane structure also enables them to evade detection and clearance by the immune system. These properties prolong their in vivo circulation half-life, promote immune activation and antigen presentation, and strengthen antitumor therapeutic efficacy [113]. Yang et al. fused 4T1 breast cancer cell membranes with lemon-derived vesicles to fabricate hybrid vesicles. This design improves vesicle stability and enables sustained drug release within the tumor microenvironment. The resulting vesicles also show low immunogenicity and circulate longer in vivo. Through homotypic targeting, these vesicles show improved tumor accumulation and enhanced chemotherapeutic efficacy [114]. Two independent studies have separately investigated heterologous recombination and homologous fusion methods. Their findings confirm that engineered reconstruction of lipid layers can serve as a practical and widely applicable method.

Surface PEGylation effectively reduces non-specific clearance and prolongs circulation. However, long-term or repeated administration may induce anti-PEG antibodies and cause accelerated blood clearance. Lipid bilayer engineering or membrane fusion enhances stability and endows new functions. This strategy achieves better stealth and targeting performance but requires stricter quality control to maintain batch consistency.

### 3.3. Drug Loading Optimization

Efficient loading is the premise for PDEVs to achieve delivery functions. Therefore, the corresponding loading method should be selected according to the different physical and chemical properties of the drug.

#### 3.3.1. Physical Loading Method

This method uses external energy to temporarily disrupt the lipid bilayer and to form transient pores for drug entry. It is particularly suitable for hydrophilic macromolecules such as siRNA, microRNA, and proteins.

Electroporation is a widely used drug loading technique that creates nanoscale pores in membranes via controlled electric field pulses and is particularly suitable for loading hydrophilic macromolecules, such as nucleic acids, into PDEVs. Although this method may cause aggregation of nucleic acid drugs, such problems can be effectively alleviated by optimizing buffer components such as trehalose and EDTA, as well as key electroporation parameters, including total vesicle count, drug-to-vesicle ratio, pulse capacitance, and electric field strength [115,116,117]. Previous studies have significantly enhanced DOX loading capacity, carrier recovery rate, and drug efficacy in vesicles through optimization of the aforementioned parameters, achieving a 190-fold increase in response compared to the free drug [82]. These findings provide critical parameter references for electroporation loading in PDEVs. In research, investigators loaded microRNA182-5p into ginseng root-derived extracellular vesicles via electroporation. The resulting N-exo-microRNA182-5p construct was shown to target and regulate the NOX4/Drp-1/NLRP3 signaling pathway, significantly improving acute lung injury in sepsis both in vivo and in vitro [16].

Sound perforation technology utilizes the cavitation effect of ultrasound to transiently disrupt the lipid bilayer, exhibiting a gentler mechanism of action than that of electroporation. A study has demonstrated the loading of curcumin (CUR) onto ginger-derived extracellular vesicles (GDEVs) via ultrasonic cultivation. The optimal conditions were 3 min of sonication and a carrier-to-drug ratio (GDEVs: CUR) of 1:1. This method achieved a high loading capacity (94.027% ± 0.094%) and encapsulation efficiency (89.300% ± 0.344%). Curcumin-loaded ginger nanovesicles demonstrated superior efficacy in improving disease activity, colon length, liver-spleen ratio, myeloperoxidase activity, and biochemical factor levels in mice with ulcerative colitis [118].

#### 3.3.2. Chemical Loading Method

Such methods rely on the chemical potential energy or intermolecular forces and are primarily applicable to hydrophobic small-molecule drugs.

PH Gradient Method: By establishing a pH gradient between the lumen (acidic) and the external environment (alkaline) of PDEVs, this method drives the active accumulation of weakly basic drugs within vesicles, achieving high loading efficiency. However, this may affect the pH-sensitive active cargo [119,120,121].

Hydrophobic Interaction Method: This approach leverages the affinity between hydrophobic drugs and the lipid bilayer of PDEVs, enabling their spontaneous incorporation into the membrane. Although this method is straightforward, excessive drug loading may lead to drug precipitation [122].

The coincubation method involves mixing PDEVs with the drug and allowing passive loading through a concentration gradient. Unloaded drugs in solution can be removed by ultrafiltration. This method is the most straightforward; however, it usually has a low efficiency and is difficult to control [123]. The passive incubation method is widely used because of its simple operation. However, loading efficiency is highly dependent on the physicochemical properties of the drug. It works better for small molecules with positive charges, but has limited efficiency for large protein molecules [96]. To enhance its efficiency, it can be combined with the active loading method. For instance, when loading HSP70 protein into tomato/grapefruit EVs, the combination of passive incubation and mild ultrasound treatment not only achieved efficient loading, but also ensured the preservation of the biocompatibility and intrinsic antioxidant activity of the EVs, thereby demonstrating the feasibility of this combined strategy.

The core of the ion-mediated loading method is to achieve loading through the electrostatic bridging effect of divalent cations and regulation of membrane properties. A study has shown that by appropriately adding Ca^2+^, the loading efficiency of 10-hydroxycamptothecin (HCPT) in clathrin vesicles can be enhanced. Unlike physical loading methods that depend on external energy or passive co-incubation that merely exploits concentration gradients, the clathrin vesicles loaded with HCPT achieved a significant inhibitory effect on tumor growth, representing an efficient and adaptable loading strategy for PDEVs. This is an efficient and adaptable loading strategy for the PDEVs [124].

#### 3.3.3. Front-Loading Strategy

In addition to conventional loading methods, a variety of innovative strategies have provided key solutions for efficient drug loading in PDEVs, demonstrating great application potential, such as engineering the donor cells before their biological occurrence, so that the vesicles secreted by them naturally accumulate target cargo drugs [125,126,127]. Mesoporous silica-derived extracellular vesicles (MDEVs) are used as carriers for nanoparticle-assisted loading. Drugs are first loaded and then incubated with PDEVs, which can achieve efficient encapsulation and protection of macromolecules such as antibodies and also give colon-targeted release function [11,128]. For the problem of oral delivery of anti-TNF-α antibodies (infliximab, INF) for IBD, the nanoparticle-assisted loading strategy provides an effective solution: large MDEVs efficiently load INF (61.3 wt%) and prevent aggregation. When co-incubated with PDEVs derived from ginger (GE, 17.5 mg kg^−1^) by ultrasound, an LMSN@GE biomimetic complex is constructed. This system exhibits gastrointestinal stability, colonic targeting, and a high intestinal epithelial permeability. Moreover, GE synergistically exerted anti-inflammatory effects by blocking the NLRP3 inflammasome. The therapeutic effect of this system in mice with colitis is significantly better than that of intravenous injection of INF after oral administration [129]. Based on the charge interaction loading technology, the surface charge of the vesicle membrane is reversibly modified by polycation, and controlled osmotic shock is combined to promote the loading of nucleic acid drugs such as mRNA. Although it may lead to a moderate increase in particle size, the loading efficiency is significantly better than the traditional passive incubation method, which provides key technical support for the development of mRNA vaccines [130].

Physical loading (electroporation, sonoporation) achieves high loading efficiency for nucleic acids and hydrophilic molecules. Yet it may temporarily disrupt membrane structure. Chemical loading (pH gradient, hydrophobic interaction) is suitable for small-molecule drugs. It is mild but shows cargo-dependent efficiency. Frontier pre-loading or hybrid-loading strategies improve scalability and uniformity, yet their regulatory compliance and standardization remain to be established.

In summary, passive targeting suffices for preliminary and safety-oriented studies, whereas active targeting is preferred for precision delivery to tumors or inflammatory sites. Surface engineering, particularly PEGylation, enhances systemic stability and prolongs circulation. Physical or chemical loading methods should be selected according to drug hydrophilicity and molecular size. In practice, a rational combination of multiple strategies is often required to balance targeting accuracy, in vivo stability, loading efficiency, and biosafety. A comparative summary of these engineering strategies, including their best-fit applications and key trade-offs, is provided in Table 3.

**Figure 4:**
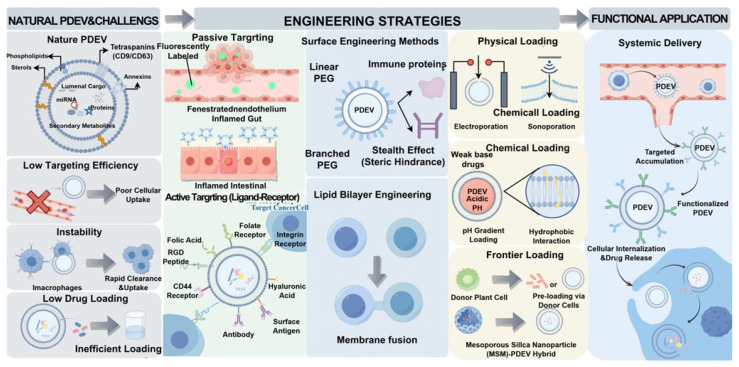
Overview of Engineering Modification Strategies for PDEVs. This figure illustrates the complete pipeline of PDEV engineering: (1) the structure and inherent limitations of natural PDEVs (low targeting, instability, and poor drug loading; the red cross (×) indicates inefficient or failed targeting and cellular uptake); (2) diverse modification strategies, including passive and active targeting, surface PEGylation, membrane fusion, physical/chemical drug loading, and frontier hybrid systems; and (3) the functional application workflow of engineered PDEVs for systemic delivery, targeted accumulation, cellular internalization, and drug release, highlighting their potential as advanced nanocarriers for precision therapy.

In summary, passive targeting suffices for preliminary and safety-oriented studies, whereas active targeting is preferred for precision delivery to tumors or inflammatory sites. Surface engineering, particularly PEGylation, enhances systemic stability and prolongs circulation. Physical or chemical loading methods should be selected according to drug hydrophilicity and molecular size. In practice, a rational combination of multiple strategies is often required to balance targeting accuracy, in vivo stability, loading efficiency, and biosafety. A comparative summary of these engineering strategies, including their best-fit applications and key trade-offs, is provided in Table 3. The engineering modification strategies discussed throughout Section 3—including passive and active targeting, surface and lipid bilayer engineering for stability, and physical, chemical, and front-loading methods—are visually summarized in Figure 4, which illustrates the complete workflow from native PDEV limitations to functional therapeutic delivery.

## 4. Performance and Synergistic Mechanisms of PDEVs as Drug Delivery Systems

The lipid bilayer and internal aqueous phase of PDEVs also confer the ability to encapsulate exogenous hydrophilic or hydrophobic molecules while protecting them from degradation [131,132]. As an innovative drug delivery platform, the core advantage of PDEVs is that they can produce a synergistic effect between their inherent therapeutic activity and the loaded therapeutic drugs, thereby reducing the dosage and minimizing side effects while enhancing the efficacy [31,133,134,135].

### 4.1. Carrier Performance of PDEVs

Before detailing the individual carrier properties of PDEVs, it is instructive to critically position them against established drug delivery nanocarriers. Compared to synthetic liposomes and polymeric nanoparticles, PDEVs offer distinct advantages in terms of renewable source availability, lower production cost, and—most notably—intrinsic bioactivity (e.g., anti-inflammatory, antioxidant, or anti-tumor effects) synergizing with loaded therapeutics rather than serving as inert delivery vehicles. Unlike mammalian exosomes, which exhibit homotypic targeting capabilities but suffer from low yield and high manufacturing costs, PDEVs can be isolated from abundant and low-cost plant biomass, making them more feasible for large-scale production. However, PDEVs also face clear limitations: batch-to-batch consistency remains a major challenge due to variations in plant sources, cultivation conditions, and extraction protocols; their drug loading capacity, particularly for hydrophilic macromolecules, is generally lower than that of synthetic liposomes; and their regulatory maturity lags significantly behind that of synthetic nanocarriers, with no PDEV-based product yet approved for clinical use. Furthermore, direct head-to-head comparative studies evaluating PDEVs versus other carriers under identical conditions remain scarce, representing a critical gap in the literature. Therefore, rather than positioning PDEVs as a universal replacement for existing platforms, this review advocates for a problem-driven selection approach: PDEVs are particularly well-suited for applications where intrinsic bioactivity is desired (e.g., anti-inflammatory therapy), low immunogenicity is critical, or oral/topical delivery routes are prioritized. In the following sections, we systematically examine the protective, targeting, and controlled-release properties of PDEVs, with an emphasis on how these capabilities compare—both favorably and unfavorably—to those of synthetic and mammalian-derived counterparts where evidence permits.

#### 4.1.1. Protective

The lipid bilayer structure of PDEVs can effectively protect nucleic acids, proteins, and small-molecule chemotherapy drugs from degradation by enzymes in the blood circulation and tissue environment. Particularly in oral delivery, it significantly enhances drug stability and bioavailability in the harsh gastrointestinal environment [136,137]. Its protective properties depend not only on the physical barrier function of the lipid bilayer but are also closely related to the inherent resistance to enzymatic degradation associated with the composition and origin of the membrane surface.

PDEVs exhibit strong protective effects in oral delivery systems, particularly for compounds that are easily degraded in the gastrointestinal tract. In a recent study, researchers developed an oral delivery system termed PEG@SN-MNs, utilizing mulberry leaf-derived extracellular vesicles that were modified with DSPE-PEG2000 and encapsulated with silymarin nanocrystals. This system significantly improves stability in gastrointestinal fluids and enables the sustained release of drugs, highlighting the potential of engineered PDEVs to enhance the oral bioavailability of small molecules [138]. In the field of nucleic acid drug delivery, PDEVs also demonstrate outstanding protective properties. For example, pine Cherry EV can protect bound microRNAs from degradation and ensure their complete arrival in the intestine after oral administration. Its distribution was detected in the small intestine, liver, and spleen. This further confirms the feasibility and effectiveness of PDEVs as oral carriers for nucleic acid drugs [139]. These studies demonstrate that the delivery system based on PDEVs can effectively overcome the gastrointestinal barrier, providing promising strategic support for the development of oral formulations of small molecules and nucleic acid drugs.

#### 4.1.2. Targeted

With ligand conjugation and similar modifications, PDEVs switch from passive to active targeting, which improves lesion accumulation and treatment efficacy [140].

PDEVs’ platforms for mRNA delivery hold considerable promise for vaccine design and development. For instance, orange juice-derived EVs have been tested as mRNA vaccine carriers. They can load mRNA encoding SARS-CoV-2 spike proteins with high efficiency. When administered orally or intranasally to mice, these vesicles trigger strong humoral and T cell immune responses, identifying plant extracellular vesicles as adaptable mRNA vaccine platforms. They have clear advantages in activating both mucosal and systemic immunity. Grapefruit-derived EVs loaded with therapeutic microRNA can cross the blood–brain barrier and accumulate in diseased brain regions. In mice bearing colorectal cancer liver metastases, these vesicles preferentially localize to tumor sites and inhibit tumor progression [141,142]. For instance, ginger-mimetic EVs loaded with 6-gingerol orally produced much higher colon drug levels and longer dwell times than free drugs, and intestinal macrophages easily took up these vesicles. This points to a promising approach for the targeted treatment of local conditions, such as ulcerative colitis [143]. These advances have pushed forward the clinical use of PDEVs in gene therapy and precision medicine.

#### 4.1.3. Controlled Release

PDEVs can achieve a slow and continuous release of drugs, which helps maintain an effective therapeutic concentration at the lesion site and avoids toxic side effects caused by sudden drug release. The release kinetics depend on the differences in drug properties and location: hydrophobic drugs embedded in the lipid bilayer are mainly released slowly through membrane diffusion, whereas hydrophilic drugs encapsulated in the cavity are mostly released through membrane degradation or endocytosis of PDEVs, thereby achieving long-term treatment [144,145].

PDEVs demonstrate multilevel delivery advantages for controlled drug release. In terms of passive controlled release, the composite system constructed using grapefruit-derived extracellular vesicles and heparin nanoparticles further enhanced the system performance through synergy. It not only improves the loading efficiency of DOX but also prolongs the circulation time of the system, thereby indirectly strengthening the long-term controlled release of the drug and its antitumor effect [146]. In the field of active controlled release, emerging light-controlled strategies have significantly expanded the ability to precisely control the timing and location. A light-responsive system based on *Pueraria lobata*-derived extracellular vesicles utilizes its endogenous photoprotective property to generate ROS in situ under specific wavelengths of light, thereby inducing a reversible permeability transition of the vesicle membrane. With an approximately 80% loading efficiency for macromolecular model drugs, this system also allows light parameters to preset membrane permeability, setting the stage for subsequent on-demand and targeted release. This strategy balances the loading efficiency and membrane integrity well, offering a way to turn PDEVs into externally controllable smart delivery platforms.

### 4.2. Synergistic Mechanisms Between Intrinsic Biological Activity and Drugs

The core advantage of PDEVs over traditional synthetic carriers is their inherent pharmacological activity, which can generate a significant synergistic effect with the drugs that they deliver. This synergy was achieved through the following three mechanisms.

#### 4.2.1. Mechanism 1: Tumor Microenvironment Modulation for Enhanced Drug Sensitivity

PDEVs can regulate the disease microenvironment through their bioactive components, reverse the pathological characteristics of the lesion, and, thereby, enhance the sensitivity of target cells to chemotherapy drugs [147,148]. When bitter melon-derived EVs were combined with 5-fluorouracil (5-FU), they not only inhibited the expression of inflammasome NLRP3 by virtue of the RNA components contained therein but also promoted apoptosis by inducing the mitochondrial ROS pathway through the MAP30 protein, thus effectively reversing chemotherapy resistance in an oral squamous cell carcinoma model and significantly enhancing the cytotoxicity of 5-FU [149]. In preclinical studies, EVs isolated from ginseng can remodel the tumor immune microenvironment by regulating macrophage polarization and inhibiting melanoma growth [150,151].

#### 4.2.2. Mechanism 2: Mitigating Drug Toxicity to Achieve “Reduced Toxicity and Enhanced Efficacy”

The anti-inflammatory and antioxidant properties of PDEVs help to reduce or prevent chemotherapy-induced organ damage and inflammation, thereby improving therapeutic efficacy and safety. In a preclinical study, EVs isolated from Rhodiola rosea were surface-modified with pYEEIE peptides and loaded with DOX. These PDEVs showed a strong tumor-targeting ability and effectively inhibited tumor growth. Notably, the inherent cardiovascular protective effects of REVs eliminated the cardiotoxicity and liver damage caused by free DOX, achieving the goal of “reducing toxicity while enhancing efficacy” [152]. Similarly, DOX-loaded celery EVs, cabbage EVs, folic acid-modified ginger EVs, and heparin-cRGD-modified lemon EVs all exerted anti-tumor effects without obvious systemic toxicity, and some carriers could even promote normal cell proliferation [94]. These results together indicate that PDEVs can effectively alleviate their toxicity and side effects while delivering chemotherapeutic drugs, providing an important path for the construction of efficient and low-toxic tumor treatment strategies.

#### 4.2.3. Mechanism 3: Multi-Pathway Parallel Attack, Synergistic Treatment

PDEVs are rich in complex bioactive components such as microRNAs, proteins, and lipids, enabling multi-target regulatory potential. Synergistic interactions with single-target chemical drugs to construct complementary action networks, enabling multidimensional synergistic treatment for complex diseases [153]. For example, *Allium tuberosum*-derived extracellular vesicles (A-DEVs) inherently exhibit anti-neuroinflammatory properties. When co-administered with the anti-inflammatory agent dexamethasone (DEX), the combined formulation (Dex-A-DEVs) significantly surpassed the efficacy of each agent individually in suppressing microglial inflammation. This demonstrates the additive and synergistic effects of natural anti-inflammatory activity and synthetic drugs acting on the same pathway [154]. Another study confirmed that cabbage-derived extracellular vesicles can be used as drug delivery carriers to load chemotherapeutic drugs such as doxorubicin. While directly killing tumor cells, its inherent anti-inflammatory and anti-apoptotic activities can simultaneously regulate the inflammatory microenvironment and cell survival signals and cooperate in multiple ways to improve the overall therapeutic effect [31].

**Figure 5:**
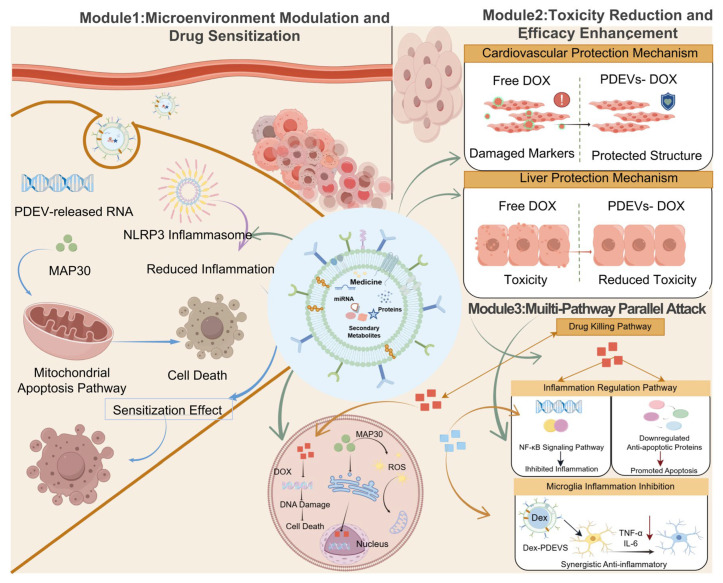
Synergistic Mechanism Between PDEVs’ Intrinsic Biological Activity and Drugs.

The three synergistic mechanisms described above are conceptually summarized in Figure 5, which illustrates how PDEVs modulate the tumor microenvironment, mitigate drug toxicity, and enable multi-pathway therapeutic attacks.

(1) They modulate the microenvironment and sensitize tumors by suppressing inflammation and inducing apoptosis. (2) Encapsulating chemotherapeutics, such as DOX, within PDEVs reduces systemic toxicity, thereby protecting cardiovascular and hepatic function. (3) Anti-tumor activity proceeds through multiple pathways: direct killing by the drug, inhibition of inflammation via NF-κB signaling, and microglia-targeted anti-inflammatory actions.

### 4.3. PDEVs-Gel Composite Systems: A Novel Platform for Local Drug Delivery

PDEV–gel composites are not simply a combination of two materials but a rationally designed delivery platform that follows clear structure–function relationships. Hydrogels do not merely provide physical retention; they actively modulate multiple key behaviors of PDEVs, including vesicle stabilization, sustained release, bioactivity preservation, membrane integrity, and cellular internalization. By matching gel properties (stiffness, porosity, degradation, responsiveness) to PDEV characteristics (membrane composition, targeting, stability, cargo), the composite system can be logically integrated into the overall PDEV engineering strategy to achieve spatiotemporally controlled, safe, and efficient local delivery.

This integrated design principle ensures that the hydrogel matrix and PDEVs work synergistically to enhance therapeutic outcomes, rather than acting as independent components. As summarized in Table 4, this rational strategy has been successfully applied in wound healing, bone regeneration, inflammation control, and local tumor therapy. A study has encapsulated EV in hydrogels and administered it orally. The results show that its gastrointestinal stability is significantly improved, and it can specifically target the intestinal mucosa, thus effectively treating IBD [155]. Zhou et al. developed a fibrin composite gel incorporating Gouqi-derived extracellular vesicles (Gq-DEVs), which can facilitate efficient delivery of PDEVs to myocardial infarction sites and improve cardiac function to reduce infarct size and myocardial fibrosis, inhibiting the p38 MAPK-NF-κB signaling pathway. It also remodels myocardial lipid metabolism, highlighting its multifunctional therapeutic potential in myocardial repair [12].

**Figure 6:**
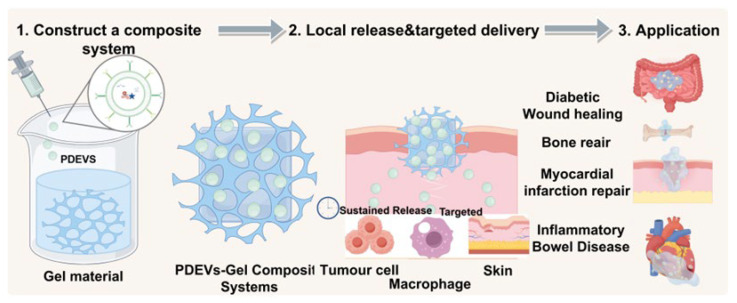
Schematic Diagram of PDEVs-Gel Composite System Construction and Application. This figure illustrates the process of the PDEV hydrogel composite biomaterial: (1) Constructing a 3D porous hydrogel system loaded with functionalized PDEV; (2) Local sustained-release and targeted drug delivery, acting on tumor cells, macrophages and skin cells; (3) Key translational applications in diabetic wound healing, bone repair, myocardial infarction repair and inflammatory bowel disease (IBD), highlighting its potential as an advanced local treatment platform.

In summary, the rational integration of PDEVs with hydrogel matrices transforms local delivery from passive retention into a controllable, spatiotemporally tunable platform. The key design principles and representative applications discussed throughout Section 4.3 are systematically summarized in Table 4 (which categorizes gel–PDEV systems by application area, gel material, mechanism, and advantages) and schematically illustrated in Figure 6 (which depicts the cascade from composite construction to local sustained release and therapeutic action). Together, these composites highlight the synergistic potential of PDEVs and hydrogels in advancing localized treatment strategies.

## 5. Challenges and Outlook

Although PDEVs demonstrate great potential for application, there are still a series of key challenges for them to move from basic research to clinical translation. This section systematically analyzes the current core bottlenecks and the key development directions for the future.

### 5.1. Current Challenges

The majority of contemporary studies on PDEVs are confined to the preclinical phase, with clinical translation impeded by several significant challenges. Firstly, the absence of standardized protocols for isolation, characterization, and quality control results in poor experimental reproducibility. Secondly, variations in plant source, cultivation, harvesting, and extraction conditions contribute to batch-to-batch variability, affecting vesicle size, composition, and bioactivity. Thirdly, the lack of established regulatory guidelines, GMP-compliant scalable production processes, standardized raw material sourcing, and comprehensive safety assessments constitutes a substantial barrier to clinical trials and commercialization. Collectively, these deficiencies significantly constrain the clinical translation of PDEVs.

Despite their therapeutic potential and low toxicity, pharmacokinetic studies are not sufficient to confirm long-term safety. Specifically, the long-term fate of plant-derived microRNAs, proteins, and other active ingredients in mammals, as well as the potential risks associated with cross-species gene regulation, have not been systematically evaluated through long-term toxicological assessments. In addition, although the commonly used PEGylation modification can prolong the circulation half-life, it may induce accelerated blood clearance and accelerate its clearance in vivo.

The on-demand and controlled release of drugs is the core goal of an ideal delivery system. However, the release behavior of PDEVs is complexly affected by multiple factors such as their membrane properties, drug properties, and lesion microenvironment. At present, there is still a lack of effective means to accurately control their release kinetics.

The synergistic mechanism between the carrier function of PDEVs and their intrinsic activity is not clear. Specifically, the extent to which the loaded drugs or other active ingredients penetrate mammalian cells and exert their effects; the mechanisms by which PDEVs are internalized following uptake, as well as their subsequent metabolism and clearance within the body; and whether the active components transported by PDEVs exhibit antagonistic or synergistic interactions with the loaded drugs.

### 5.2. Future Direction

Future research should prioritize the following directions:

Using synthetic biology to transform plant cells and increase the production of PDEVs by regulating related genes, transform the metabolic pathway to enrich more active ingredients in PDEVs, or directly introduce targeting molecules into cells so that PDEVs have natural targeting ability, thereby eliminating tedious post-modification steps.

Promoting the innovation and standardization of separation and purification technology is the key to realizing the scale of PDEVs. For example, the development of continuous flow microfluidic devices to achieve high-throughput separation, lectin affinity chromatography, and other techniques was used to improve purity and yield. Optimize the combined process of tangential flow filtration and size exclusion chromatography to achieve stable and uniform large-scale production under the premise of ensuring vesicle activity [168].

Integrating systems biology and artificial intelligence methods to comprehensively uncover the mechanisms of action and regulatory networks of PDEVs. For example, integrating transcriptomics, proteomics, lipidomics, and metabolomics to systematically identify their active substances; combining live-cell imaging and single-particle tracking techniques to elucidate their intracellular transport and drug release processes; and using artificial intelligence to deeply explore the intrinsic relationships between data [169].

**Figure 7:**
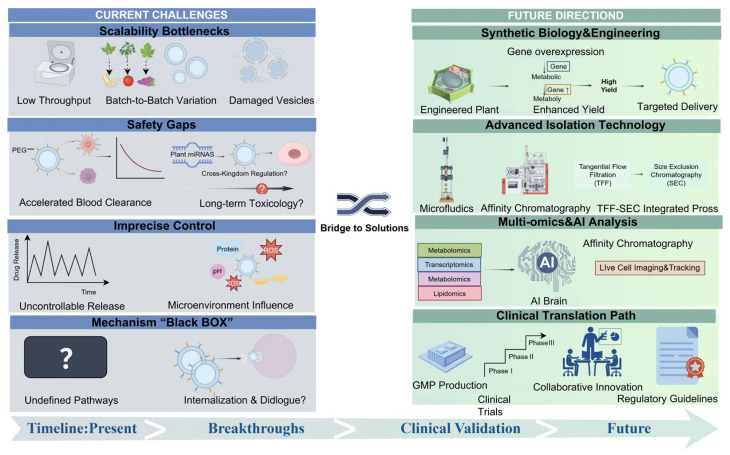
Schematic Diagram of PDEVS Challenges and Future Outlook. This figure summarizes the main limitations and unresolved issues in current PDEV research, including accelerated blood clearance, imprecise drug release control, and poorly defined biological mechanisms (question marks indicate unresolved scientific questions, such as cross-kingdom regulation, long-term toxicology, and undefined internalization pathways). Corresponding solutions and future directions are also proposed, covering advanced purification technologies, multi-omics and artificial intelligence analysis, and standardized clinical translation pathways to bridge the gap between basic research and clinical application.

To maximize the clinical value of PDEVs, further translational research and a sound regulatory system are urgently needed. Feasible production protocols complying with GMP guidelines should be established first, with strict standardization of raw material sourcing and plant cultivation to reduce batch variation, while refining manufacturing workflows to move beyond lab-scale preparation and achieve viable large-scale production. On this basis, clinical trials should be arranged rationally for conditions with unmet clinical needs, focusing on systematic evaluation of safety, pharmacokinetic performance, and preliminary therapeutic outcomes in human subjects. At the same time, academic researchers, regulatory bodies, and industry partners need to work together to formulate unified technical standards for PDEV characterization, quality control, and safety assessment so as to clarify the regulatory classification and approval procedures for these plant-derived vesicles. Improving regulatory arrangements, standardizing raw material supply, realizing GMP-compliant scalable production, and adopting standardized clinical trial evaluation criteria will jointly provide a solid foundation for the clinical translation and future practical application of PDEVs. The major challenges and proposed future directions discussed in this section are visually summarized in Figure 7, providing a concise overview of the translational roadmap for PDEVs.

## 6. Conclusions

PDEVs possess potential for clinical application in drug delivery, as they combine biocompatibility with intrinsic bioactivity that distinguishes them from synthetic nanocarriers. This review summarizes the core engineering strategies for optimizing PDEVs, including targeted modification to enhance tissue-specific delivery, stability improvement, and optimized drug loading, as well as the innovative application of hydrogel integration to address local delivery challenges. Despite these advancements, several critical challenges remain unresolved, including the lack of standardized protocols for isolation, purification, and quality control, batch-to-batch variability arising from raw material differences, underdeveloped regulatory frameworks, and gaps in translating preclinical findings to clinical practice. To effectively address these issues, an integrated, interdisciplinary approach—rather than isolated solutions—is essential, involving collaboration across research fields to establish unified quality standards, scalable manufacturing processes, and clear regulatory guidelines. Future research should focus on refining GMP-compliant production systems, standardizing clinical trial designs for unmet medical needs, and leveraging advanced technologies to bridge the gap between laboratory research and clinical application, thereby fully unlocking the clinical potential of PDEVs as a transformative nanotherapeutic platform within precision medicine.

## Figures and Tables

**Table 1:** Analysis of Sources, Characteristics, Application Potential, and Engineering Prospects for Representative PDEVs.

Organ	Plants	Isolation & Purification Methods	Core Bioactivity	Therapeutic Applications	Key Features & Engineering Prospects	References
root	Ginseng	Ultracentrifugation (UC), Density Gradient Centrifugation (DGC)	Antioxidant; anti-inflammatory; cardioprotective; anti-tumor	Inflammatory Bowel Disease (IBD); sepsis-induced Acute Lung Injury (ALI); diabetic oral ulcers; glioma inhibition	Edible-medicinal; readily available high cost; blood–brain barrier penetration; gel-compatible; high phospholipid membrane	[15,16,17,18,19,20]
	*Pueraria lobata*	Differential Centrifugation (DC)	Anti-alcoholic, antioxidant, anti-inflammatory; autophagy/gut microbiota regulation	Alcohol-related disorders, osteoporosis, rheumatoid arthritis	Edible-medicinal, readily available; targeting ability; drug loading/gel composite compatible; high biosafety	[21,22,23,24]
stem	Ginger	UC	Anti-inflammatory; antioxidative; anti-tumor	Intestinal inflammation; obesity-induced Non-Alcoholic Fatty Liver Disease (NAFLD); osteoarthritis	Edible-medicinal; highly available, low cost; microRNA/Nrf2 pathway regulation; gut microbiota modulation; drug loading/targeting modification	[25,26,27,28]
	Yam	DC; 100,000× *g* UC	osteoblast formation promotion	stimulate osteoblast formation and prevent osteoporosis	Edible-medicinal, high biosafety; osteogenesis-targeted, potential for bone disease delivery system development	[29]
leaf	*Perilla frutescens*	UC	Anti-inflammatory; antioxidative (*Pab-miR-396a-5p*)	Psoriasis alleviation (IL-17 signaling modulation)	Edible-medicinal; readily available, medium cost; microRNA/drug loading; excellent biocompatibility; potential for inflammatory diseases	[30]
	Cabbage	UC and size-exclusion chromatography; Ultrafiltration (UF)	Anti-inflammatory; anti-proliferative; small noncoding tsRNA encapsulation	Postinjury arterial restenosis prevention	Edible; readily available, low cost; high yield; tsRNA/drug loading compatible; isolation method versatile	[31,32,33]
flower	Honeysuckle (*Lonicera japonica*)	Tangential Flow Filtration (TFF)	Anti-inflammatory; gut microbiota modulation; ROS response	Postoperative abdominal adhesion, IBD, MAFLD, acute liver failure protection	Edible-medicinal, readily available; ROS-responsive hydrogel compatible; drug loading potential; high biosafety	[34,35,36,37]
	Edible tea flowers	UC, DGU	anti-metastatic	Metastatic breast cancer suppression	Edible, readily available; high biosafety; anti-tumor potential; suitable for anti-cancer drug delivery development	[38]
fruit	Grape	DC, DGU, UC	Anti-inflammatory, antioxidative, anti-vascular calcification; anti-photoaging	DSS-colitis alleviation, vascular calcification, skin photoaging, LPS/D-GalN-induced acute liver failure	Edible; readily available, low cost, high yield; oral delivery suitable; drug loading/surface modification compatible	[39,40,41]
	Tomato	UC	Anti-inflammatory; wound healing; anti-tumor	Calcitriol delivery for enhanced anti-cancer effect; renal cyst inhibition	Edible-medicinal; low cost; large-scale production feasible	[42,43,44,45]

**Table 2:** Comparison of Extraction Methods, Principles, Properties and Applications for PDEVs.

Extraction Method	Extraction Principle	Suitable Plant Materials	Advantages	Disadvantages	Combination Strategy & Applications	References
UC	Sedimentation rate differences under centrifugal force	Various clarified plant juices and cell culture supernatants	process large sample volumes	High equipment cost; time-consuming.	UC + SEC: Proteomics/mechanism research	[61,62]
DGC	Vesicle flotation speed & equilibrium density	Clarified plant juices; cell culture supernatants	High separation precision; low protein contamination	labor-intensive and time-consuming, specialized equipment	UC + DGC: Precise biochemical analysis	[63]
UF	Size-based separation	Large-volume; simple-composition plant extracts	processing large amounts of plant material a	it is insufficient for eliminating small impurities	UF + SEC: Functional research/therapeutic candidate preparation	[64]
SEC	Particle size difference	Clear, low viscosity plant extracts	Preserves vesicle integrity; high yield/recovery; short time	not eliminate protein contaminants of a similar size to PDEVs	UF/TFF + SEC: Therapeutic grade purification	[65,66]
Flow Field-Flow Fractionation (FlFFF)	Nanoparticle size-based separation	Large-volume; viscous plant materials; fine-particle samples	Maintains particle size uniformity; high resolution	Suitable for pre-purified samples only	UC/TFF + FlFFF: High-resolution particle size analysis	[67,68]
TFF	Membrane pore size separation; tangential flow anti-blockage	Large-volume; viscous plant materials; fine-particle samples	Scalable; high activity recovery	High initial equipment investment; membrane adsorption loss	TFF + SEC: Clinical translation	[69]
Immuno-affinity Separation (IAS)	Surface biomarker-specific binding	PDEVs subpopulations with specific membrane proteins	Improve specificity and purity	Limited by reliable surface marker availability.	Post-DGC/SEC refining: Targeted vesicle subpopulation isolation	[70]
Polymer-Based Precipitation (PBP)	According to the difference in solubility in PEG	Crude; complex; high-viscosity extracts	processing large sample volumes	Low purity; requires additional purification	Precipitation + UC/SEC: Rapid vesicle capture for functional studies	[71]

**Table 3:** Comparative Overview of PDEV Engineering Strategies.

Strategy Category	Specific Approach	Best Suited For	Key Advantage	Limitation/Trade-Off
Targeting (Passive)	Source selection and administration route	Preliminary studies; safety-focused development	Simple; preserves PDEV natural activity	Low specificity; depends on EPR effect or tissue tropism
Targeting (Active)	Ligand conjugation (folate, RGD, aptamer, antibody)	Precise delivery to tumors or inflammatory sites	High specificity	Requires chemical modification; may alter surface properties
Stability (Surface engineering)	PEGylation; membrane cloaking	Systemic delivery requiring prolonged circulation	Reduces nonspecific clearance; extends half-life	Risk of ABC phenomenon upon repeated dosing
Stability (Lipid bilayer engineering)	Cholesterol enrichment; phospholipid remodeling; membrane fusion	Oral or topical delivery where stability in harsh environments is needed	Enhances resistance to enzymatic degradation; preserves intrinsic bioactivity	More modest half-life extension compared to PEGylation
Loading (Physical)	Electroporation; sonication	Hydrophilic macromolecules	High loading efficiency	May cause vesicle aggregation or cargo degradation
Loading (Chemical)	pH gradient; hydrophobic interaction; coincubation	Small hydrophobic drugs	Gentle; preserves vesicle integrity	Lower efficiency for hydrophilic drugs
Loading (Front-loading)	Donor cell engineering; nanoparticle-assisted; charge interaction	Challenging cargos (antibodies, mRNA, combination therapies)	High loading for difficult cargos	Less standardized; requires specialized expertise; high complexity

**Table 4:** Applications and Design Strategies of PDEVs-Gel Composite Systems in Local Drug Delivery.

System	Gel Material	PDEVs Source	Mechanism	Advantages of Gel Plant-Derived EVs Complex	Application	References
Diabetic Wound Repair	GelMA/DAS Hydrogel	Lemon	Macrophage-mediated immune regulation; angiogenesis/fibrosis promotion	Tissue-adhesive; moist/breathable; sustained PDEVs release	Repairing diabetic wounds	[156]
	GelMA-Dopamine Hydrogel (MEMC-Gel)	*Momordica charantia*	Resists inflammation and oxidative stress, promotes fibroblast migration and angiogenesis, and regulates macrophage polarization.	Favorable mechanical properties; biocompatible; degradable	Diabetic wound healing and repair	[157]
	Microenvironment-responsive collagen hydrogel	*Houttuynia cordata Thunb*	NF-κB/YAP pathway regulation; angiogenesis/collagen deposition enhancement; anti-inflammation	Tissue-adhesive; self-healing; antioxidative/anti-inflammatory	Diabetic wound repair	[158]
	Hydrogel	Mango ginger	Promotes keratinocyte migration via induction of FSTL1 protein.	Regulates the wound immune microenvironment; excellent transdermal capability.	Rescued delayed wound healing caused by diabetes	[159]
Infection/Inflammation-Related Wound Repair	silk protein methacryloyl hydrogel	Broccoli	NF-κB pathway inhibition; anti-inflammatory macrophage polarization; antimicrobial/antioxidative activity	High biocompatibility; MRSA/E. coli killing; ROS scavenging	Promotes anti-scarring healing of MRSA-infected wounds	[160]
	ROS-responsive DNA hydrogel	Ginseng	Wound microenvironment modulation; inflammatory/immune pathway downregulation; energy metabolism upregulation	Controlled G-Exos/NO release; microenvironment-responsive	Infected wound healing	[161]
	CDENs-based hydrogel	Coriander	Nrf2 pathway activation; antioxidant enzyme defense system enhancement; anti-inflammation	Sustained CDENs-release and excellent biocompatibility.	Wound healing acceleration	[162]
Bone Tissue Repair	Gelatin Methacryloyl	lotus roots	ROS scavenging; TGF-β/Smad pathway regulation; osteogenesis-angiogenesis coupling; bone marrow mesenchymal stem cells recruitment	High biocompatibility; sustained LREVs release; multifunctional synergy	Stimulate osteogenesis-angiogenesis coupling for accelerated bone regeneration	[163]
	3D GelMA hydrogel	Epimedium	PI3K/Akt pathway activation; osteogenic marker upregulation (RUNX2, TGF-β1, etc.)	improve PDEVs retention, stability, and sustained release	Bone tissue engineering	[164]
Tumor Therapy	Dual-functional injectable adhesive hydrogel	Ginger	Hemostasis; ROS scavenging; anti-inflammation; targeted tumor elimination	combines hemostasis, antioxidation, tissue repair, and recurrence prevention.	Osteosarcoma recurrence suppression; post-resection wound healing	[165]
	Modified HA-CMCS injectable hydrogel, in situ crosslinked via Schiff base reaction	*Panax ginseng* root; spinach	Tumor apoptosis induction; PD-L1 downregulation; MHC-I upregulation; CD8^+^ T cell activation; TME remodeling	sustained GENs release; strong tumor retention; synergistic effects; easy preparation	Solid tumor immunotherapy (especially TNBC); immunochemotherapy combination	[166]
Mucosal Inflammation Treatment	Semi-IPN Hydrogel	*Rabdosia rubescens*	promoted collagen deposition, targeted NLRP3 to reduce inflammation	targeted therapy, improve bioavailability, and efficient healing	Healing of Chemoradiotherapy-Induced Oral Mucositis	[167]
Myocardial Repair	Fibrin gel	Gouqi (*Lycium barbarum*)	p38 MAPK/NF-κB p65 pathway inhibition	increased the delivery efficiency of GqDNVs	Cardiac repair after myocardial infarction	[12]

## Data Availability

The original contributions presented in this study are included in the article. Further inquiries can be directed to the corresponding author.

## Abbreviations

The following abbreviations are used in this manuscript:

5-FU	5-fluorouracil
A-DEVs	*Allium tuberosum*-derived extracellular vesicles
ALI	Acute Lung Injury
BPNs	Bacterial-plant hybrid membrane vesicles
cRGD	Cyclic RGD
CUR	Curcumin
DC	Differential Centrifugation
DEX	Dexamethasone
DGC	Density Gradient Centrifugation
DOX	Doxorubicin
EVs	Extracellular vesicles
EXPO	Extracellular vesicle-positive organelle
FlFFF	Flow Field-Flow Fractionation
GDEVs	Ginger-derived extracellular vesicles
Gq-DEVs	Gouqi-derived extracellular vesicles
GSH	Glutathione
HCPT	Hydroxycamptothecin
IAS	Immuno-affinity Separation
IBD	Inflammatory Bowel Disease
ICG	Indocyanine green
ILVs	Intraluminal vesicles
INF	Infliximab
MDEVs	Mesoporous silica-derived extracellular vesicles
MLEs	Mulberry leaf–derived extracellular vesicles
MPS	Mononuclear phagocyte system
MVB	Multivesicular body
NAFLD	Non-Alcoholic Fatty Liver Disease
NTA	Nanoparticle Tracking Analysis
PBP	Polymer-Based Precipitation
PDEVs	Plant-derived extracellular vesicles
PEG-ACNVs	PEGylated nanovesicles derived from *Asparagus cochinchinensis*
ROS	Reactive oxygen species
SEC	Size exclusion chromatography
TEM	Transmission electron microscopy
TFF	Tangential Flow Filtration
UC	Ultracentrifugation
UF	Ultrafiltration
UPA	Urokinase-type plasminogen activator
